# Clinical characteristics and outcomes of patients with H1N1 influenza pneumonia admitted at a tertiary care hospital in Karachi, Pakistan

**DOI:** 10.1186/s41479-020-00070-7

**Published:** 2020-07-05

**Authors:** Mujahid Hussain, Nosheen Nasir, Muhammad Irfan, Zahra Hasan

**Affiliations:** 1 Department of Medicine, Section of Pulmonary and Critical Care Medicine, Aga Khan University, Karachi, Pakistan; 2 Department of Medicine, Section of Infectious Diseases, Aga Khan University, Karachi, Pakistan; 3 Department of Pathology and Laboratory Medicine, Section of Molecular Pathology, Aga Khan University, Karachi, Pakistan

**Keywords:** Influenza, H1N1, Mortality

## Abstract

**Introduction:**

Influenza viruses specifically, A and B mainly contribute to seasonal outbreaks that occur globally. However, due to limited diagnostics for influenza there is little data regarding clinical outcomes of patients with H1N1 pneumonia in our region. Our objective was to determine the clinical characteristics and outcomes of patients hospitalized with H1N1 pneumonia at a tertiary care facility in Karachi, Pakistan.

**Methods:**

A retrospective study of adult patients admitted with influenza pneumonia from November 2017 to February 2018 at a tertiary care hospital in Karachi, Pakistan. Patient characteristics were compared between influenza A H1N1 and other types of influenza using multivariable logistic regression analysis and subgroup analysis for factors associated with mortality in H1N1 Pneumonia was performed.

**Results:**

Out of 497 adult patients with community acquired pneumonia (CAP), 172 fulfilled the criteria for Influenza like illness (ILI). 88 patients had PCR confirmed Influenza pneumonia of whom *n* = 57 (65%) had Influenza A H1N1. The mean age of patients 53.5 years (SD: 17.3) and 60% were male. The overall mortality from Influenza in this study was 15.9% (*n* = 14); out of these 11 (78.5%) had Influenza A H1N1. Multivariable analysis showed that the increase in length of hospital admission was significantly associated with H1N1 Influenza A infection (OR: 1.47 CI: 1.2–1.8). Factors associated with mortality showed that presence of ARDS, Septic shock and multi-organ failure was highly significantly associated with death (*p*-value < 0.001) along with deranged liver function tests (*p*-value 0.01) and presence of nosocomial infection (*p*-value 0.027).

**Conclusion:**

Influenza A H1N1 is associated with greater length of stay compared with infection due to other types of Influenza and mortality in H1N1 Pneumonia was found to be associated with presence of nosocomial infection among several other factors which may have implications given higher rates in a low-middle income country.

## Background

Viral respiratory infections account for nearly 50% of acute respiratory illness. Influenza viruses A and B cause seasonal outbreaks with significant morbidity and mortality. The first influenza A (swine flu) virus of the H1N1 subtype was isolated from in humans in 1974 [1]. In 2009, the World Health Organization (WHO) announced a swine-oriented influenza virus pandemic, H1N1 2009 pandemic [2]. During this outbreak in 2009/2010, around 18,500 deaths were reported associated with confirmed H1N1 influenza virus with a mortality rate of 0.12 deaths per 100,000 populations [3]. According to United States Centers for Disease Control and Prevention (CDC), there were 12,470 fatal cases based on data using mathematical models [4]. A majority of patients with Influenza A H1N1 subtype usually present with symptoms of upper and lower respiratory tract infections such as, sudden onset of fever, cough, sore throat, headache, myalgia, and generalized weakness. The symptoms may be self-limiting in most but it can progress to severe pneumonia, acute respiratory distress syndrome (ARDS) and mortality [5, 6]. Studies have shown that up to 66% hospitalized patients had chest radiographic abnormalities compatible with pneumonia [3]. There are no unique clinical features that distinguish Influenza A subtype H1N1 infection from other forms of viral respiratory infections. Although a number of patients had predisposing immune-compromising conditions, healthy persons are also at risk for illness and death from H1N1 Influenza pneumonia [2]. In a study from Turkey; 30.6% patients presented with interstitial infiltrates on chest radiograph and 36% with consolidation [7]. Studies have shown increased severity of disease in pregnant women, transplant recipients and patients with malignancies [8, 9]. There have been conflicting data from different parts of the world with some regions reporting better survival in ventilated patients [10] and some regions with poor outcomes in patients requiring intensive care [11].

Influenza A H1N1 has been reported globally but there is limited data available from Pakistan. In 2009, a single center case series reported 36 confirmed H1N1 cases with 16.7% mortality from in Pakistan [12]. Most of these cases were previously healthy individuals who developed an influenza-like illness that progressed to pneumonia within 5 to 7 days (5).

Despite yearly outbreaks each winter; data available from Pakistan on clinical characteristics and outcomes of H1N1 pneumonia patients is scarce [12, 13] and none is available from recent outbreaks. The aim of the study was to determine the clinical characteristics, course of disease and outcomes of these patients when hospitalized with H1N1 pneumonia in tertiary care hospital.

## Methods

### Ethical approval

This study received approval from the Ethical Review Committee of the hospital (5277-Med-ERC-18). The data was collected from hospital records and the requirement for informed consent was waived by the hospital ethical review committee as data was anonymized and no personal identifiers were collected.

### Study population and design

We conducted a retrospective descriptive study of adult patients admitted with a diagnosis of Influenza pneumonia from November 2017 to February 2018 at a 700 bedded tertiary care hospital in Karachi, Pakistan. All patients admitted with the diagnosis of community acquired pneumonia during the study period were identified from the hospital information management system. Influenza-like illness (ILI) was defined as “An acute respiratory illness with a measured temperature of ≥38 °C and cough, with onset within the past 10 days” [14]. A confirmed case of influenza pneumonia was defined as an individual with an ILI with laboratory-confirmed influenza by Influenza virus PCR assay in respiratory specimen.

Demographic and clinical characteristics of confirmed influenza patients including, underlying medical and respiratory co-morbidities and laboratory and radiological investigations, treatment and complications during hospitalization were collected on a structured performa from hospital medical records.

### Diagnosis of influenza

Nasopharyngeal swabs were processed for detection of Influenza virus by real-time reverse transcriptase polymerase chain reaction (RT-PCR) using the Influenza virus PCR assay (Altona Diagnostics, GmbH).

Radiological diagnosis of pneumonia was made by evaluation of infiltrates observed on chest X ray and/or CT chest scans. A multidisciplinary team of doctors including infectious diseases consultants, pulmonologists and intensivits were involved in identification of cases and their management.

### Data analysis

STATA ver 12.1was used for data analysis. Descriptive analysis was performed for demographic features with mean and standard deviation reported for quantitative variables such as age and lengths of hospital stay and frequencies (percentage) for qualitative variables such as gender, co-morbid conditions, mortality and complications etc. χ2 test of independence or Fischer Exact test was performed for comparison of factors between those with H1N1 Influenza compared to those with Influenza other than H1N1 subtype. Multivariable logistic regression analysis was performed on variables found to be significant on univariate analysis to identify factors associated with Influenza A H1N1 infection. All *p* value ≤0.05 was taken as significant. Subgroup analysis was also done using χ2 test of independence or Fischer Exact test for comparison between those who died compared to those who recovered from H1N1 Influenza.

## Results

Total of 497 patients of age 18 years and above were admitted during this period with community acquired pneumonia (CAP) out of which 172 fulfilled the criteria for Influenza like illness (ILI). Out of 172 patients admitted with ILI, 88 (51%) patients fulfilled the criteria of confirmed Influenza pneumonia. Majority of the patients had Influenza A H1N1 (*n* = 57) 65% while, (*n* = 6) 7% patients had non H1N1 Influenza A and (*n* = 25) 28% patients had Influenza B.

The mean age of patients with H1N1 Influenza was 53.5 years (SD: 17.3). Thirty-four patients (59.6%) were female and six of them were pregnant. Hypertension was the commonest co-morbid condition as found in 44% of H1N1 cases. Sixty-three percent of the Influenza A H1N1 patients were non-smokers. Only three (5.2%) patients had received quadrivalent inactivated influenza vaccination.

Risk factors associated with influenza were evaluated in patients with H1N1 influenza. None of the cases had a recent history of travel outside of the country or, had occupational exposure to livestock. Only one was a health care provider. The median duration of illness prior to presentation of Influenza was 5 days (IQR: 2) (Table 1). Amongst Influenza A H1N1 patients, 96% patient had interstitial infiltrates on chest radiography and the most frequently associated lung condition was Chronic Obstructive Pulmonary disease (COPD) (*n* = 8). Of patients with Influenza A H1N1 subtype, microbiological cultures of respiratory secretions taken on admission were tested in 23 cases and were found to be positive in 6 (26%) patients. Three patients had a polymicrobial growth of *Staphylococcus aureus* and *Aspergillus* species (two had *A. flavus* and one had *A. fumigatus*) and three patients had isolated growth of *Aspergillus flavus*. All patients with H1N1 influenza received Oseltamivir as well as antibiotics empirically.

**Table 1:** Baseline characteristics of patients with Influenza subtype H1N1 compared to other than H1N1 during the outbreak of 2017

Characteristic	H1N1 (*N* = 57)	Other than H1N1 (*N* = 31)	*p*-value
**Mean Age ± SD**	53.5 ± 17.3	61.3 ± 12.6	0.035
Mean CURB score ± SD	1.9 ± 0.98	1.5 ± 0.99	0.091
Median Duration Of Illness (Range)	5 (2–10)	4 (2–7)	0.636
**Gender**			0.467
Male	23	15	
Female	34	16	
Pregnant	6	0	0.001
**Smoking status**			1.000
Current Smoker	7	4	
Ex-Smoker	11	6	
Non Smoker	36	20	
Don’t know	3	1	
**Co-morbids**			
Diabetes	24	12	0.757
Hypertension	25	18	0.203
COPD	8	4	
Cardiac Problems	13	3	
Obesity	5	1	
CVA	2	0	
Malignancy	2	1	
Immunosuppression	7	2	

Abbreviations: *COPD* Chronic Obstructive Pulmonary disease; *CVA* Cerebrovascular Accident

The clinical outcomes of patients with Influenza A H1N1 infection are summarized in Table 2. The mean length of stay was 8 days (SD: 6). The overall mortality from Influenza virus infection during this period was 15.9% (14/88). Eleven (78.5%) of 14 patients who died were positive for Influenza A H1N1 subtype. Further, the mortality in Influenza A subtype H1N1 pneumonia was 19% (11/57).

**Table 2:** Outcomes and Complications of patients with Influenza subtype H1N1 compared to other than H1N1 during the outbreak of 2017

Variables	H1N1 (N = 57)	Other than H1N1 (N = 31)	*p*-value
**Type of admission**			
ICU	19	0	0.001
SCU	28	17	0.968
Ward	10	13	0.013
**Management**			
Vasopressors	13	1	0.016
Non-invasive ventilation	20	9	0.564
Invasive Ventilation	19	0	< 0.001
Mean Duration Of Intubation ± SD	3.06 ± 4.97	0	
Systemic Steroids	23	11	0.654
Antibiotics	56	29	0.282
Oseltamivir	57	28	0.041
**Complications**			
Respiratory Failure	34	9	0.009
ARDS	18	0	< 0.001
Septic Shock	14	1	0.015
MODS	13	3	0.157
Nosocomial Infection	7	0	0.049
Acute Kidney Injury	24	6	0.032
NSTEMI	9	2	0.315
**Outcome**			0.359
Recovered	42	26	
Dead	11	3	
Unknown	4	2	
Median Length Of Stay (Range)	6 (1–25)	3 (0–12)	0.001

Abbreviations: *ICU* Intensive care unit; *SCU* Special care unit; *ARDS* Acute Respiratory Distress Syndrome; *MODS* Multi-organ dysfunction Syndrome; *NSTEMI* Non- ST elevation myocardial infarction

The laboratory parameters including mean white blood cell count, creatinine and blood glucose levels were similar for both patients with H1N1 and influenza other than H1N1 with the exception of transaminase levels (SGPT and SGOT) which had a higher trend in patients infected with Influenza A subtype H1N1 pneumonia (Supplementary Table 1).

Amongst patients with H1N1, nineteen (33.3%) required ICU admission and mechanical ventilation whilst none of the other cases (with influenza A non-H1N1 or Influenza B), *p*-value: 0.001. The mean duration of intubation was 3.06 days (SD: 4.97) among 19 patients, who required mechanical ventilation. This was significantly different when compared to patients who had influenza other than H1N1 as none of patients in that group required mechanical ventilation. The mortality among mechanically ventilated patient were 47.3% (*n* = 9/19). Twenty nine (33%) patients required non-invasive ventilation (NIV). Twenty of 29 patients who required NIV had H1N1 pneumonia. Five patients who required NIV died and the association was not statistically significant (*p* = 0.7); 4 of these patients had H1N1 pneumonia.

The hospital course was complicated by nosocomial pneumonia in 8 (14%) of those with H1N1 influenza compared to none in the other group (*p*-value: 0.046) (Table 2). Patients were diagnosed to have superimposed infection based on culture and sensitivity results of sputum or bronchoscopy specimens obtained during course of hospital stay. *Acinetobacter* species was isolated in 5 patients and *Pseudomonas aeruginosa* was isolated in 3 patients. *Aspergillus* species were identified from sputum specimens of 8 patients, 6 at the time of admission and 2 during the course of hospital stay. *Aspergillus flavus* was the predominant species in 6 out of 8 specimens. 4 patients in whom Aspergillus species had been isolated had H1N1 pneumonia, one had Influenza A non- H1N1 and remaining had Influenza B pneumonia. None of these patients required ICU admission and only one patient died.

Associated factors with H1N1 Influenza A compared to other Influenza types were assessed using univariate and multivariate analysis. In univariate analysis, presence of ARDS and invasive ventilation were found to be highly significant (*p* = value < 0.001). Other significant risk factors included intensive care unit admission (*p*-value: 0.001), septic shock (*p*-value: 0.015), respiratory failure (*p*-value: 0.009) and increase in length of stay (*p*-value: 0.001). However in the multivariable analysis, only increase in length of stay was significantly associated with H1N1 Influenza infection (OR:1.47 CI: 1.2–1.8) and increase in age was found to be protective for H1N1 infection (OR:0.93 CI: 0.89–0.97) suggesting that H1N1 may be more predominantly affecting relatively younger age groups after adjusting for confounding.

Subgroup analysis for factors associated with mortality in H1N1 influenza was done using Fischer Exact test and it was found that presence of ARDS, Septic shock and multi-organ failure was highly significantly associated with death (*p*-value < 0.001) along with deranged liver function tests (*p*-value 0.01) and presence of nosocomial infection(*p*-value 0.027) (Table 3).

**Table 3:** Subgroup analysis for mortality in H1N1 pneumonia

Variables	Died (*n* = 11)	Recovered (*n* = 42)	*p*-value
Invasive ventilation	9	9	< 0.001
Respiratory failure	10	20	0.001
ARDS	10	7	< 0.001
Septic Shock	10	4	< 0.001
MODS	9	2	< 0.001
Nosocomial Infection	4	3	0.027
Transaminitis	7	6	0.010

Abbreviations: *ARDS* Acute Respiratory Distress Syndrome; *MODS* Multi-organ dysfunction Syndrome

## Discussion

The World Health Organization (WHO) declared Influenza H1N1virus to have transitioned into post-pandemic period but with the potential to cause outbreaks in 2010. WHO Surveillance reports from December 2017 showed predominant circulation of influenza A(H1N1)pdm09 virus with influenza A(H3N2) in Pakistan. Our study is the first from the country to describe the clinical characteristics and outcomes of patients affected with Influenza in that period. As per CDC, Influenza season between 2017 and 2018 was of high severity with multiple people visiting emergencies as well outpatient clinics with Influenza like illness and deaths due to pneumonia and influenza were reported to be approximately 10% nationally across the United States with predominant circulating strain being Influenza A(H3N2) [15]. As opposed to this, in Europe, the majority of cases were due to influenza virus type B infection with excess mortality from all causes as per European Centre for Disease Prevention. However, from South Asia including, Afghanistan and India, the predominant strain was H1N1 [16]. In a single center study from India, the mortality was 29% but it did not include cases from the 2017 outbreak. In our study, overall mortality from Influenza was 15.9% and mortality from H1N1 subtype was 19%. Study from Pakistan sentinel sites reported 11% mortality from H1N1 in 2009 [13] and 16% in another single center study [12]. Mean age in our cohort was 55 years as opposed to 36 years during the 2009 Influenza outbreak in Pakistan [13]. However gender distribution also reflected similar predilection for females [12]. Among the risk factors, we did not find significant association with smoking or obesity as reported from other studies [17, 18] and majority of our patients were non-smokers as opposed to another study from Pakistan [13]. Eighteen percent required ICU admission as compared to 27% in another study done during the 2009 pandemic [12].

Secondary bacterial pneumonia is a well-recognized complication of influenza and most commonly implicated organisms include *Staphylococcus aureus Streptococcus pneumonia and Klebsiella sp*. [19]. In our study we found Aspergillus species in 6 out of 23 sputum specimens sent at admission with 3 having concomitant *Staphylococcus aureus*. *S. aureus* has been reported to cause superimposed infection in patients with H1N1 influenza [20, 21]. However, Aspergillus species have recently been reported to complicate severe influenza infections [22]. Influenza associated Aspergillosis (IAA) has been reported to occur in immunocompetent patients with Influenza and a large multicenter study from Netherlands reported 16% IAA in their cohort. They also concluded that approximately all cases that have been reported so far are associated with influenza A H1N1 infection [23]. However, the limitation of our study is that we could not differentiate colonization with *Aspergillus* from Influenza associated Aspergillosis. Moreover our study has limited generalizability as it’s a single center study. Findings from this study have implications for management in developing countries where the burden of multi-drug resistant healthcare associated infections and invasive fungal infections is high [24, 25] and may adversely affect outcomes in Influenza A H1N1 pneumonia since mortality is greater in patient who developed nosocomial infections.

## Conclusion

Influenza A H1N1 is associated with greater length of stay compared with infection due to other types of Influenza and mortality in H1N1 Pneumonia was found to be associated with presence of nosocomial infection among several other factors which may have implications given higher rates in a low-middle income country.

## Supplementary information

**Additional file 1: Supplementary Table 1.** Laboratory Data for H1N1 pneumonia patients compared to Influenza other than H1N1. Abbreviations: WBC: White blood cell count; CRP: C-reactive protein; SGPT: Serum glutamic pyruvic transaminase.

## Data Availability

All data generated or analysed during this study are included in this published article.

## Abbreviations

WHO: World Health Organization
CDC: Centers for Disease Control and Prevention
ARDS: Acute respiratory distress syndrome
ERC: Ethical Review Committee
ILI: Influenza-like illness
RT-PCR: Real-time reverse transcriptase polymerase chain reaction
CAP: Community acquired pneumonia
COPD: Chronic Obstructive Pulmonary disease
SGPT: Serum glutamic pyruvic transaminase
SGOT: Serum glutamic-oxaloacetic transaminase
ICU: Intensive care unit
NIV: Non-invasive ventilation
IAA: Influenza associated aspergillosis
