# Supplementary material for: Clinical characteristics and outcomes of patients with H1N1 influenza pneumonia admitted at a tertiary care hospital in Karachi, Pakistan
Source: Pneumonia (Nathan). 2020 Jul 5;12:5. doi: 10.1186/s41479-020-00070-7 (PMC7335362; doi:10.1186/s41479-020-00070-7)
Supplement: Supplementary file 1 — Additional file 1: Supplementary Table 1. Laboratory Data for H1N1 pneumonia patients compared to Influenza other than H1N1. Abbreviations: WBC: White blood cell count; CRP: C-reactive protein; SGPT: Serum glutamic pyruvic transaminase. [file 41479_2020_70_MOESM1_ESM.docx]

Supplementary Table 1: Laboratory Data for H1N1 pneumonia patients compared to Influenza other than H1N1

| Laboratory test | H1N1 | Influenza other than H1N1 |
| --- | --- | --- |
| HEMOGLOBIN g/dl (mean± SD) | 11.79 ± 2.20 | 12.58 ± 2.04 |
| HEMATOCRIT (vol%) (mean± SD) | 36.08 ± 6.20 | 38.13 ± 5.95 |
| WBC x 10^9^ cells per liter (mean± SD) | 10.23± 6.94 | 10.47 ± 6.19 |
| NEUTROPHILS %(mean± SD) | 77.34 ± 14.94 | 74.96 ± 16.55 |
| BLOOD GLUCOSE mg/dl (mean± SD) | 152.30 ± 77.64 | 165.5 ± 75.90 |
| CRP mg/L (mean± SD) | 11.20 ± 7.85 | 11.12 ± 7.74 |
| PROCALCITONIN ng/ml (mean± SD) | 5.85 ± 12.44 | 1.83 ± 3.32 |
| UREA mg/dl (mean± SD) | 25.93 ± 24.23 | 18.62 ± 8.50 |
| CREATININE mg/dl (mean± SD) | 3.97 ± 18.33 | 1.12 ± 0.39 |
| SODIUM Meq/L (mean± SD) | 131.74 ± 21.30 | 134.03 ± 21.27 |
| BICARBONATE Meq/L (mean± SD) | 22.44 ± 5.30 | 23.01 ± 5.97 |
| TOTAL BILIRUBIN mg/dl (mean± SD) | 0.98 ± 1.14 | 0.45 ± 0.18 |
| SGPT mg/dl (mean± SD) | 71.87 ± 82.53 | 31.75 ± 19.04 |

Abbreviations: WBC: White blood cell count; CRP: C-reactive protein; SGPT: Serum glutamic pyruvic transaminase
